# Application of WALANT technique for repairing finger skin defect with a random skin flap

**DOI:** 10.1186/s13018-021-02319-3

**Published:** 2021-03-02

**Authors:** Jianhua Xu, Lu Yin, Shuming Cao, Haihua Zhan, Jianbing Zhang, Qiang Zhou, Ketong Gong

**Affiliations:** Department of Hand Microsurgery, Tianjin Hospital, NO. 406, South Jiefang Road, Hexi District, Tianjin, 300211 People’s Republic of China

**Keywords:** Wide-awake local anesthesia no tourniquet; Surgical flaps; Fingers; Hand injuries

## Abstract

**Background:**

Wide-awake local anesthesia no tourniquet (WALANT) technique has emerged among hand surgeons with other indications. Surgeries involving pedicled flap and revascularization are no longer used as contraindications. The present study aimed to evaluate the feasibility and merits of the WALANT technique in random skin flap surgery.

**Methods:**

From May 2018 to March 2019, 12 patients with finger skin defects repaired with random skin flaps were reviewed. Abdominal skin flaps or thoracic skin flaps were used to cover the wound. Both the fingers and the donor sites were anesthetized by the WALANT technique. A 40-mL conventional volume consisted of a mixture of epinephrine and lidocaine. A volume of 5 mL was injected at the distal palmar for nerve block, the other 5 mL was injected around the wound for hemostasis, and the remaining was injected at the donor site of flaps for both analgesia and hemostasis. Baseline data with respect to sex, age, side, type of finger, donor sites, flap size, dosage of anesthetics, usage of finger tourniquet, intraoperative and postoperative pain, hemostasis effect, operation time, Disabilities of the Arm, Shoulder, and Hand Questionnaire (QuickDASH) score, and hospitalization expense, were collected.

**Results:**

All patients tolerated the procedure, and none of them needed sedation. Single finger skin defect in 8 patients and double finger skin defect occurred in 4 patients; 5 patients were repaired by abdominal skin flaps, and 7 patients were repaired by thoracic skin flaps. The good surgical field visibility was 91.7%. All flaps survived adequately, without necrosis, pulling fingers out, and other complications. The average visual analog scale (VAS) score of the maximal pain was 1.1 in fingers vs. 2.1 in donor sites during the operation. On postoperative day one, the average VAS score of the maximal pain in fingers and donor sites was 1.3 and 1.1, respectively. The average hospitalization expense before reimbursement of the whole treatment was 11% less expensive compared to the traditional method. The average QuickDASH score was 9.1.

**Conclusions:**

Under wide-awake anesthesia, patients have the ability to control their injured upper extremities consciously, avoiding the complications due to pulling flap pedicles. With the merits of safety, painlessness, less bleeding, and effectivity, the WALANT technique in random skin flaps is feasible and a reliable alternative to deal with finger skin defect.

## Background

The brain enables humans to imagine fantastic worlds, and the hand makes man’s imagination come true. Hand injury occurs commonly among upper extremity traumas that cause large physical disabilities in patients and large workload for surgeons and large medical consumption annually for the government [1]. Hence, how to facilitate rehabilitation, simplify surgical procedure, and reduce further medical consumption, is a problem that needs to be resolved urgently. Recently, the wide-awake surgery under local anesthesia without tourniquet and sedation (WALANT) technique could achieve this goal based on the merits of simplicity, good outcomes, and cost-efficiency. This technique has become more and more popular in hand surgeons and has also been accepted by foot and ankle and plastic surgeons [2, 3]. The surgeons schedule their work time efficiently as the effect of anesthesia was activated. With the active motion of the hand in the awake patient, surgeons could make rectification in a timely manner, thus improving the postoperative outcomes [4, 5]. Not only surgeons but also patients and the government obtained hypostatic benefit from the WALANT technique [6–9].

However, in the early twentieth century, most hand surgeons avoided using epinephrine in fingers for fear of necrosis. Some literature about finger necrosis propagated the myth that epinephrine could lead to finger loss [10, 11]. For a long duration, this dogma influenced many surgeons and medical students, and using epinephrine in fingers has been regarded as the contraindication even in some professional textbooks. Until the twenty-first century, it has been demonstrated that this prejudice without sufficient evidence [12, 13]. Nonetheless, the myth dissipated following some large-sample studies that utilized epinephrine in hand and finger surgeries [14–16]. Although flap and revascularization surgeries through wide-awake anesthesia have been estimated safe [17, 18], the WALANT technique in the field of flap surgery is yet not applied widely.

Surgeons in the Hand Microsurgery Department of Tianjin Hospital (Tianjin, China) have utilized the WALANT technique since 2016. Herein, we attempted to combine this technique with random skin flap surgery for both analgesic and hemostatic effects. For these surgeries, the postoperative immobilization of the injured arm is essential, and a fully awake patient who is able to control his or her upper limb consciously to ease the procedure. Thus, we deem specific advantages of the WALANT technique in random skin flap because there is less possibility of pulling the flap pedicles for a patient whose upper limb is not anesthetized, and complications, such as swelling, ischemia, and necrosis could be avoided. Therefore, from May 2018, we commenced using the WALANT technique in random skin flap surgeries for repairing the finger skin defect. The current study demonstrated another preponderance of the WALANT technique and provided further evidence of the safety of this technique in flap surgeries. This study, for the first time, described random skin flap surgery under wide-awake anesthesia.

## Materials and methods

### Research design and objects

This retrospective study described the cases using random skin flap to repair finger skin defect by WALANT technique and analyzed the feasibility and merits of this method. From May 2018 to March 2019, 12 finger skin defect patients, who accepted random skin flap surgeries under wide-awake anesthesia, were included in this study. The patients were treated in the Hand Microsurgery Department at Tianjin Hospital. Each patient underwent two surgeries (the first stage was flap harvested surgery, and the second was pedicle division surgery).

### Inclusion and exclusion criteria

The inclusion criteria were adults with finger skin defects repaired by abdominal or thoracic random skin flaps, under wide-awake anesthesia at both the injured fingers and the donor sites. The exclusion criteria were as follows: minors, patients with palm or wrist skin defect, the donor site that was not abdomen and thorax, repair by free flaps or perforator flaps, and surgeries performed under general anesthesia or brachial plexus block or other regional block or local anesthesia with lidocaine only.

### Data collection

The basic information, such as sex, age, side, and type of finger, was collected according to the medical record. The donor sites and the size of flaps, the dosage of anesthetics, and the usage of finger tourniquet were referred by the operation notes. The operation time and the hospitalization expense (before insurance reimbursement) were referred by the nursing record and settlement list, respectively. The duration of the operation is defined as the time from completing the sterile preparation to finishing suture. The hospitalization expense included examination, anesthesia, operation, bed, and drug cost of the two surgical stages. Based on the visual analog scale (VAS) score of 0–10, patients were asked to rate the maximal pain of both the fingers and the donor sites (0 as no pain and 10 as maximal pain), and surgeons were required to rate a hemostasis effect score of the donor sites (0 as minimal bleeding and 10 as profuse bleeding) during the operation. Moreover, on postoperative day one, the VAS score of maximal pain of both the fingers and donor sites was rated by the patients. The highest level of pain between the first and the second phase of surgeries was considered as maximal for statistical analysis.

### Anesthesia technique

**Fig. 1:**
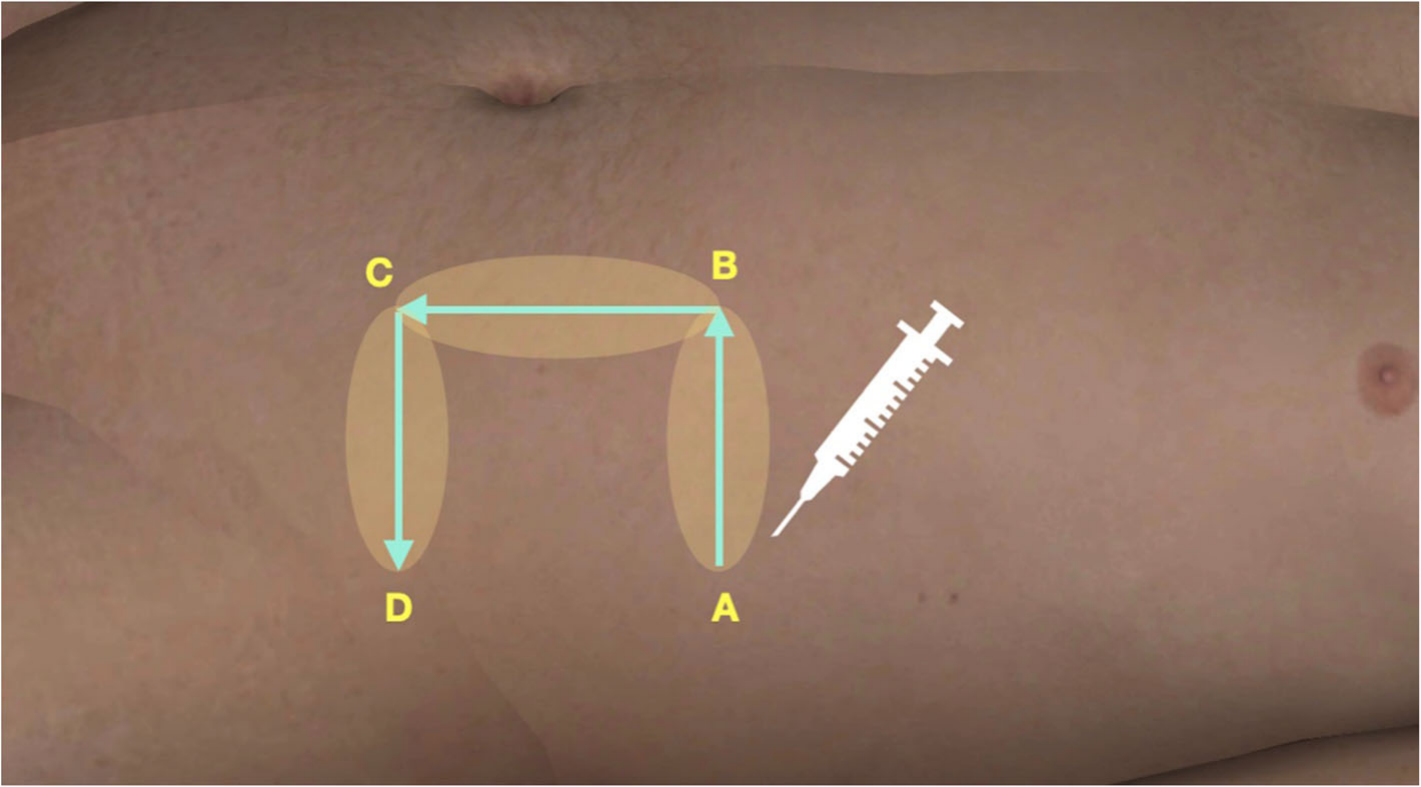
Anesthesia method: injection begins from point **a** to points **b** and **c**, then ends at point **d**, making tumescent infiltration to ensure that the needle is under the dermis and behind the tumescent area

Anesthesia was administered by the surgeons who were attending or senior doctors, and anesthesiologist was no need in this study. The lidocaine (2%) containing epinephrine (0.1%) was prepared for anesthesia injection (the concentration of epinephrine was 1:100,000). A 40-mL cocktail volume is the conventional volume for each procedure, consisting of 20 mL lidocaine, 20 mL normal saline, and 0.4 mL adrenaline. In case a large volume of solution is needed, another 40 mL normal saline is added into the conventional solution, among which the epinephrine concentration is 1:200,000 and below the maximum dose of lidocaine (7 mg/kg). At the palmar of metacarpal-phalangeal joint of each finger, 5 mL solution was injected for digital nerve block and another 5 mL was injected around the wound for hemostatic effect. At the donor site of the flap, 30–60 mL anesthetic was sufficient for injection at either the abdomen or the thorax for both analgesia and hemostasis. As the flap is usually designed as “U” shape, the first injection site was located at the beginning, and the final injection site was located at the end of the alphabet U (Fig. 1). The slow consecutive subcutaneous infiltration ensured that the needle does not go beyond the tumescent skin at any time. Surgeries begin at 5 min after anesthesia, following sterile preparation.

### Surgical technique

**Fig. 2:**
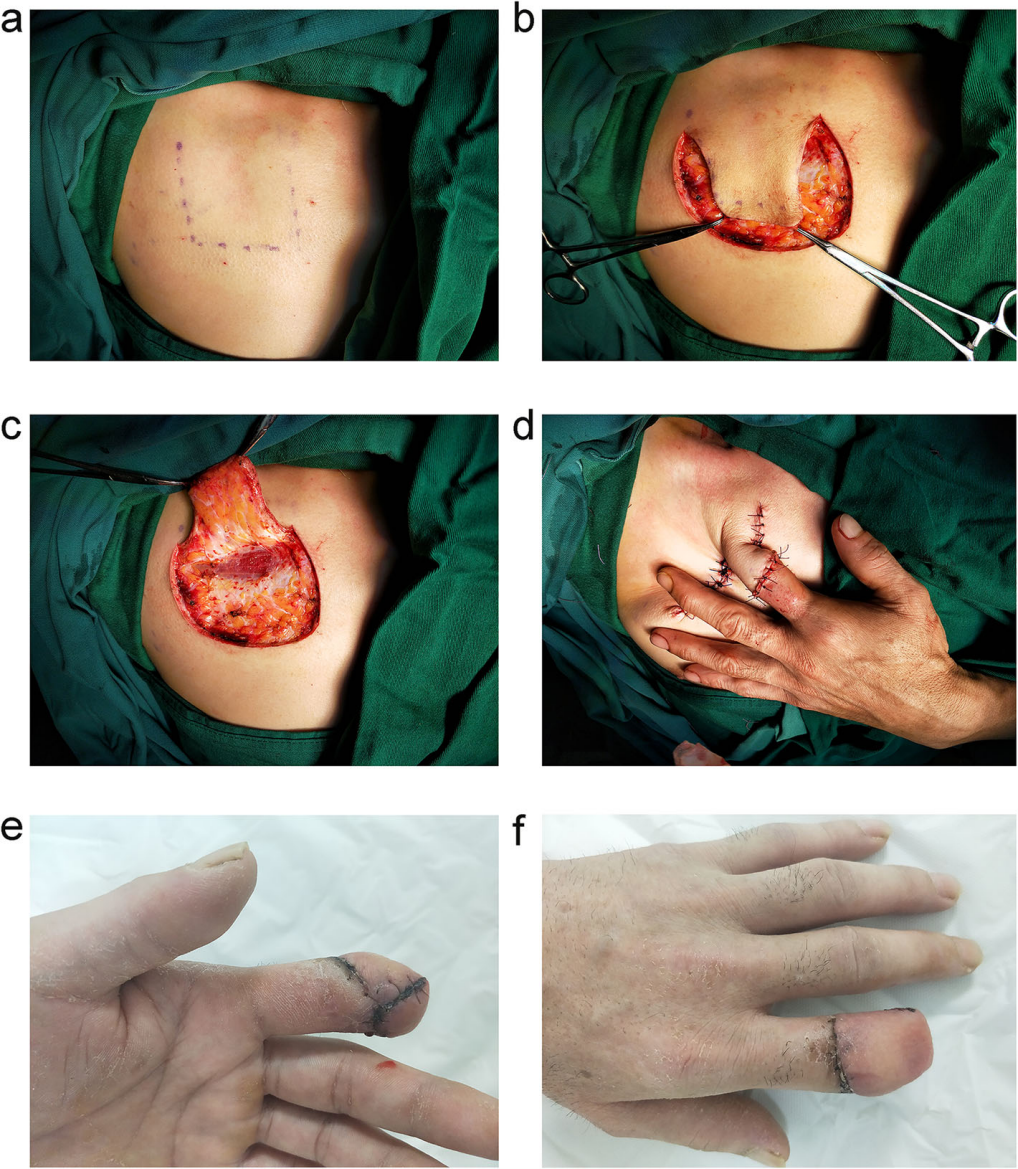
A patient whose distal phalangeal was avulsed amputation with middle phalangeal skin defect of the index finger that was repaired with a thoracic skin flap. **a** Pale skin after anesthesia; **b** incision with less bleeding; **c** elevating the flap with good field visibility; **d** covering the wound of the finger stump with flap; **e**‑**f** flap survived well after surgery

**Fig. 3:**
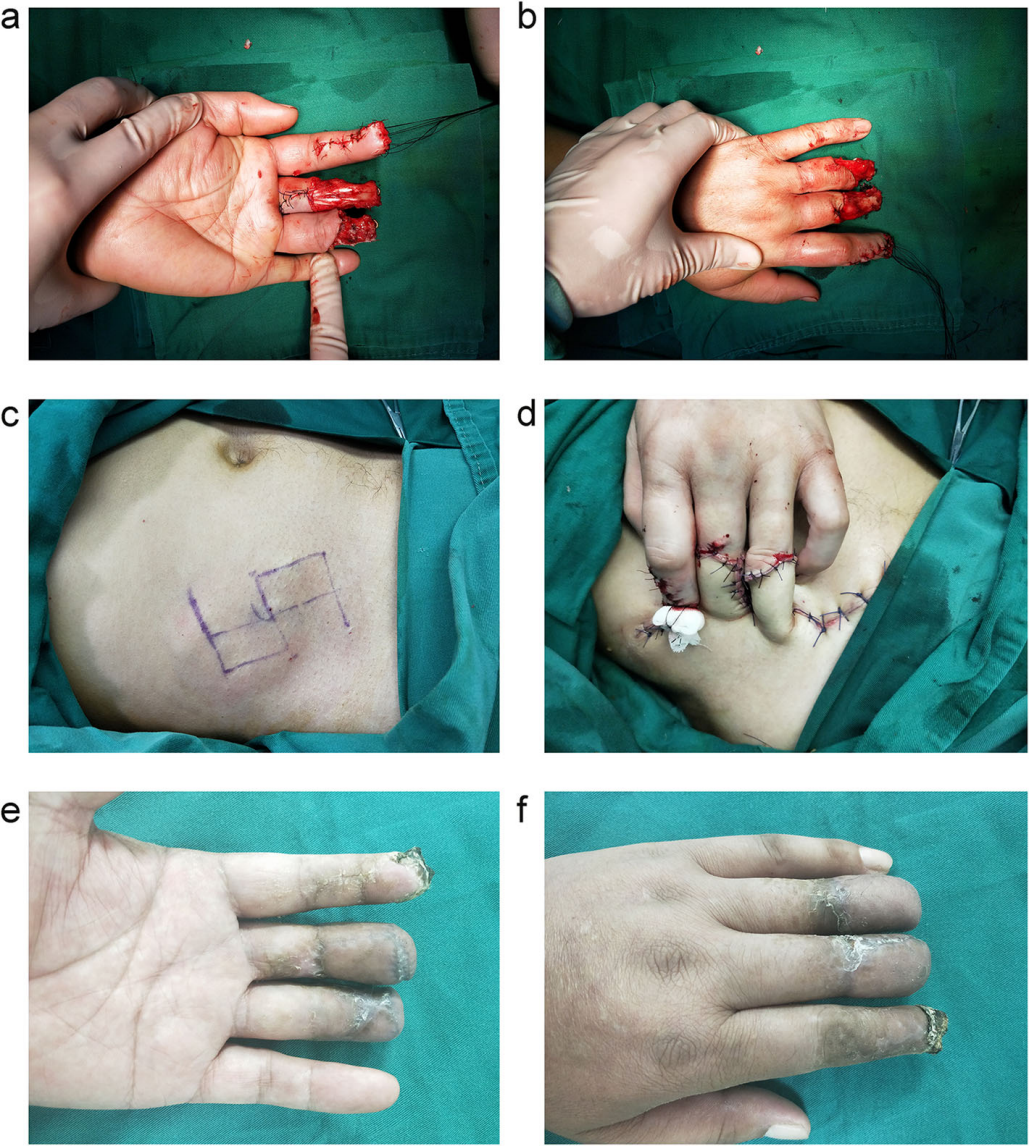
A patient whose distal phalangeal was avulsed amputation with middle phalangeal skin defect of the middle and ring finger that was repaired with abdominal skin flaps. **a**‑**b** Skin defect of the middle and ring finger stump; **c** pale skin after anesthesia and incision design; **d** covering the wound of finger stump with flaps; **e**‑**f** flaps survived well after surgery

The surgeries were performed by the attending or senior surgeons. Although an arm tourniquet is not necessary, a rubber band for the finger is useful at the initial stage of the procedure for identifying the essential structures. Firstly, thorough debridement and hemostasis were performed, and bone and joint internal fixation and soft tissue repair were carried out if necessary. Then, the size and the site of flaps were designed and selected according to the specific configuration of the skin defect. If the size of the skin defect is small, the thorax is the optimal donor site. On the other hand, if the patient is female or the size of the skin defect is large, the abdomen is the first choice. A single leaf flap is sufficient when the skin defect is a simple wound, and the flaps should be designed as a double-leaf or tubular when there is degloved injury and enlarged about 20%. The incision was in accordance with the trace of anesthesia injection, and flaps were elevated at the superficial layer of the superficial fascia. The injured hand was placed at the contralateral abdomen or thorax, and the flaps were used to cover the area of the skin defect and make sutures (Figs. 2, Fig. 3). Finally, the upper limb was bound up using elastic bandages to ensure that the pedicle was relaxed. Postoperative intravenous transfusion treatments, such as improving circulation, anti-infection, and pain control, are conventional. Subsequently, flap pedicle division was performed under wide-awake anesthesia (with the same anesthesia solution and injection method) 4 weeks after the first surgery.

### Follow-up

All patients were followed up by telephone and WeChat (a mobile text, voice, and video messaging communication service developed by Tencent in China), and the average follow-up time was 9.2 (range, 6–14) months postoperatively. Adverse events, such as pulling fingers out of flap and flap necrosis, were recorded. Complications, such as infection, wound nonunion, and subcutaneous hematoma, was noted in each patient. According to the picture and video messages from WeChat, and some characteristics, such as edema, redness, exudation, and necrosis of the skin, could be useful for assessing the wound. The shortened version of the Disabilities of the Arm, Shoulder, and Hand Questionnaire (QuickDASH) was sent to each patient and was retrieved after it was filled out.

## Results

All patients, except one, were men, with an average age of 38.9 (range, 18–60) years. The injury was in the left hand in 6 patients and right hand in 6 patients, while single finger skin defect and double finger skin defect was detected in 8 and 4 patients, respectively. Five patients were repaired by abdominal skin flaps and 7 by thoracic skin flaps; the minimal size of the single flap was 3.5 cm × 2.5 cm, and the maximal size was 8 cm × 6 cm. The average volume of anesthetic injection was 42.1 (range, 25–70) mL. Intraoperatively, three cases utilized a finger tourniquet, and the average VAS score of the maximal pain was 1.1 in fingers vs. 2.1 in the donor sites. On postoperative day 1, the average VAS score of maximal pain in fingers and donor sites was 1.3 and 1.1, respectively. In terms of hemostasis effect, the score of the donor sites was 0–10 (0 as minimal bleeding and 10 as profuse bleeding), 8 cases got score 1, 2 cases got score 2, 1 case got score 3, and 1 case got score 4. Based on the hemostasis effect score, the surgical field visibility was grouped into good (0–3), moderate (4–6), and poor (7–10), thus achieving 91.7% of good field visibility. The average operation time was 65.9 and 43.4 min of the two surgical stages, respectively. The average hospitalization expense before insurance reimbursement of the whole treatment was 48,701.2 RMB, about 11% (6,000 RMB) less than that of the traditional method (brachial plexus block of the injured upper limbs and epidural anesthesia of the donor sites), which could be saving as the need for an anesthesiologist was eliminated. Strikingly, all the flaps survived well without adverse events and complications, such as necrosis, infection, hematoma, wound nonunion, and pulling finger out of the flap. The average QuickDASH score was 9.1 (range, 0–20.5) of the total cases by the latest follow-up. The detailed information of patients is listed in Table 1.

**Table 1:** Baseline characteristics of enrolled patients

Number	Sex	Age (years)	Side	Finger	Donor site	Size of single flap (cm×cm)	Number of flaps	Finger tourniquet	Volume of anesthetic (mL)	VAS score of maximal pain				Hemostasis effect score (donor site)	Operation time (minutes)		Hospitalization expense (RMB)	QuickDASH score
										*Intraoperative* (*finger*)	*Intraoperative* (*donor site*)	*Postoperative day 1* (*finger*)	*Postoperative day 1* (*donor site*)		*1*^*st*^ *surgery*	*2*^*nd*^ *surgery*		
**1**	Female	35	Right	M	Abdomen	6×4	1	No	30	1	2	2	1	1	89	33	50000.1	0
**2**	Male	42	Left	I	Thorax	5×5	1	No	30	1	2	1	1	1	50	25	45042.0	13.6
**3**	Male	25	Right	I,M	Abdomen	8×6 4×4	2	No	60	2	4	2	2	3	72	61	60905.1	11.4
**4**	Male	44	Left	M,R	Abdomen	6×4 4×3 4×3	3	Yes	65	2	3	3	2	4	100	69	48047.1	15.9
**5**	Male	18	Right	I	Thorax	6×6	1	No	35	1	0	0	1	1	42	28	48909.2	6.8
**6**	Male	60	Right	M,R	Abdomen	5×3 4×3 4×3	3	Yes	60	1	2	1	1	2	61	55	51118.1	15.9
**7**	Male	50	Left	T	Thorax	4×4	1	No	30	1	2	1	0	1	55	35	39083.0	4.5
**8**	Male	54	Left	T	Thorax	3.5×2.5	1	No	25	0	1	1	0	1	40	30	45087.8	6.8
**9**	Male	47	Right	T	Thorax	4×3 4×3	2	No	35	0	3	2	1	1	60	41	48072.5	4.5
**10**	Male	31	Left	I	Thorax	4×3 4×3	2	No	35	1	2	1	0	1	52	35	52111.8	9.1
**11**	Male	29	Right	T,I	Abdomen	8×6 4×3 4×3	3	Yes	70	2	2	1	4	2	120	79	55015.0	20.5
**12**	Male	32	Left	L	Thorax	4×4	1	No	30	1	2	1	0	1	50	30	41023.4	0

“T” as thumb, “I” as index finger, “M” as middle finger, “R” as ring finger, and “L” as little finger

## Discussion

The method of compounding epinephrine and anesthetic for anesthesia injection is dated back to 1920s [10]. Typically, due to the vasoconstriction effect, epinephrine was added into procaine for hemostasis during the operation, but tissue necrosis was reported [11]. Therefore, epinephrine was blamed for this phenomenon as it could decrease the blood supply of the tissues. When lidocaine was formulated, the mixture of epinephrine and lidocaine rarely resulted in tissue necrosis. Then, following the advent of phentolamine, the antidote to reverse vasoconstriction gradually alleviated the epinephrine-related fear of the surgeons [6, 12, 13, 19]. Since the early twenty-first century, Lalonde et al. reported wide-awake hand surgery systemically and published the first professional book about the WALANT technique [6, 14, 19–24]. To date, this technique has been utilized by many hand surgeons [25, 26] and accepted by patients [27–29]. Flap and revascularization surgeries were no longer as contraindications of wide-awake anesthesia, as the safety and efficiency of the WALANT technique have been verified through clinical practice [17, 18].

The major advantages of WALANT technique using in hand surgery are as follows: (1) no need for fasting and excessive examination before surgery; (2) no sedative and relative side effects, such as nausea and vomiting; (3) avoiding injury of nerve and vessel that might be due to brachial plexus block; (4) eliminating discomfort caused by tourniquet, and procedures could be performed smoothly without the limit of tourniquet time; (5) patients who are fully awake to cooperate with surgeons, thereby facilitating intra-operative communication and strengthening postoperative rehabilitation confidence; (6) operation field is clear with less bleeding; (7) active flexion and extension of the fingers is preserved, which allows to check whether there is gap after tendon repairment, tendon block caused by pulleys, cross fingers, and the fracture is firm enough; (8) without postoperative recovery, patients could go home themselves and come back to work earlier; (9) achieving analgesia for a prolonged duration without increasing the usage of analgesics; (10) saving medical resource and decreasing the hospitalization expense; (11) patient satisfaction, safety, and efficiency [6, 20, 28, 30–34].

The traditional method of harvesting abdominal or thoracic skin flaps is carried out under epidural anesthesia or local anesthesia with lidocaine and normal saline only in our department, without a tourniquet; thus, excessive bleeding is inevitable during the operation. Nevertheless, wide-awake anesthesia effectuates the operation field with less bleeding, which is attributed to the vasoconstrictive effect of adrenaline. In the current study, 91.7% of cases attained good surgical field visibility. This becomes convenient for designing and trimming flaps, while it makes the hemostasis procedure simple and reduces the operation time. Under wide-awake anesthesia, the pain was acceptable, and all patients tolerated the surgery, and none of them needed sedation. The average VAS score of maximal pain was 1.1 in fingers and 2.1 in donor sites during the surgery and 1.3 in fingers and 1.1 in donor sites on postoperative day 1, which was similar to that in the previous reports [35]. Although the recommended optimal time of the hemostatic effect of epinephrine was about 30 min [36], we did not wait that long before surgery and began manipulation about 5 min after anesthesia just when sterile preparation was completed. Consequently, we found that the bleeding was acceptable in the initial surgery period, as described previously [25, 37]. We did not require an arm tourniquet, but a rubber band for the finger was sufficient in some cases. The injured limb was not paralytic and has the ability of active movement. Therefore, patients could control their upper limbs consciously and adjust the position of the injured hand when necessary, thus ensuring that the flaps were in the optimal position and the subsequent complications from pulling flap pedicles were avoided. Therefore, we deem that preserving an independent limb is the best superiority of the WALANT technique in random skin flap surgery than other anesthetic methods. No skin necrosis was detected in our cases, thus providing reliable evidence on the safety of using epinephrine in flap surgeries. In addition, this technique was cost-effective as an anesthesiologist was not required to monitor the anesthesia-related care; hence, about 11% of the whole cost could be saved on each patient of our hospital [38, 39].

Anesthesia and surgery involve the following steps: (1) thorough debridement, hemostasis, and firm fixation of fractures. A finger rubber band is used for rapid and straightforward manipulation, and if combined with tendon rupture, such flaps are selected cautiously as the risk of tendon re-rupture; (2) the injured hand is placed on the contralateral abdomen or thorax by choosing the most comfortable position, and then the site and the size of the flap was designed; (3) the anesthetic was injected at each site of the incision; however, the injection should be avoided at the flap pedicle, and the number of pinpricks should be minimal; (4) a 30-min wait before surgery is not required. The operation could begin immediately after anesthesia and sterile preparation, and the bleeding of the donor site is acceptable; (5) flaps must be enlarged about 20% and designed as a single leaf, double leaf, and tubular flap if necessary, the ratio of length to width should not be beyond 1.5:1; (6) the wounds were covered with flaps, followed by suturing with non-absorbable sutures. Elastic bandages were used to bind the injured upper limb with truncus with appropriate tension; (7) postoperative education needs to be strengthened to guide patients on maintaining the suitable position of the injured hand and managing the wound.

The limitations of this study include retrospective design that causes bias in the information, a small amount of samples, and a lack of control trial.

## Conclusions

In summary, a fully awake patient with an independent upper limb is the most specific merit of the WALANT technique in random skin flap surgery. Our practice demonstrated that using random skin flap to repair finger skin defects under the WALANT technique is feasible with the merits of safety, painlessness, and effectivity for clinical application.

## Data Availability

The datasets used and/or analyzed during the current study are available from the corresponding author on reasonable request.

## Abbreviations

WALANT: Wide-awake local anesthesia no tourniquet
VAS: Visual analog scale
QuickDASH: The shortened version of the Disabilities of the Arm, Shoulder, and Hand Questionnaire
